# Diversity and Structure of Diazotrophic Communities in Mangrove Rhizosphere, Revealed by High-Throughput Sequencing

**DOI:** 10.3389/fmicb.2017.02032

**Published:** 2017-10-18

**Authors:** Yanying Zhang, Qingsong Yang, Juan Ling, Joy D. Van Nostrand, Zhou Shi, Jizhong Zhou, Junde Dong

**Affiliations:** ^1^CAS Key Laboratory of Tropical Marine Bio-resources and Ecology, Guangdong Provincial Key Laboratory of Applied Marine Biology, South China Sea Institute of Oceanology, Chinese Academy of Sciences, Guangzhou, China; ^2^Tropical Marine Biological Research Station in Hainan, South China Sea Institute of Oceanology, Chinese Academy of Sciences, Sanya, China; ^3^Department of Microbiology and Plant Biology, Institute for Environmental Genomics, University of Oklahoma, Norman, OK, United States; ^4^University of Chinese Academy of Sciences, Beijing, China

**Keywords:** mangroves, microbial community, diazotrophs, *nifH*, high-throughput sequencing, sulfate-reducing bacteria

## Abstract

Diazotrophic communities make an essential contribution to the productivity through providing new nitrogen. However, knowledge of the roles that both mangrove tree species and geochemical parameters play in shaping mangove rhizosphere diazotrophic communities is still elusive. Here, a comprehensive examination of the diversity and structure of microbial communities in the rhizospheres of three mangrove species, *Rhizophora apiculata*, *Avicennia marina*, and *Ceriops tagal*, was undertaken using high**-**throughput sequencing of the 16S rRNA and *nifH* genes. Our results revealed a great diversity of both the total microbial composition and the diazotrophic composition specifically in the mangrove rhizosphere. *Deltaproteobacteria* and *Gammaproteobacteria* were both ubiquitous and dominant, comprising an average of 45.87 and 86.66% of total microbial and diazotrophic communities, respectively. Sulfate-reducing bacteria belonging to the *Desulfobacteraceae* and *Desulfovibrionaceae* were the dominant diazotrophs. Community statistical analyses suggested that both mangrove tree species and additional environmental variables played important roles in shaping total microbial and potential diazotroph communities in mangrove rhizospheres. In contrast to the total microbial community investigated by analysis of 16S rRNA gene sequences, most of the dominant diazotrophic groups identified by *nifH* gene sequences were significantly different among mangrove species. The dominant diazotrophs of the family *Desulfobacteraceae* were positively correlated with total phosphorus, but negatively correlated with the nitrogen to phosphorus ratio. The *Pseudomonadaceae* were positively correlated with the concentration of available potassium, suggesting that diazotrophs potentially play an important role in biogeochemical cycles, such as those of nitrogen, phosphorus, sulfur, and potassium, in the mangrove ecosystem.

## Introduction

Mangroves are unique intertidal ecosystems along tropical and subtropical coastlines and play an essential role in maintaining sea levels and protecting coasts in tropical and subtropical regions (Duke et al., 2007). In tropical marine environments, mangroves are thought to be important as primary producers of organic matter, providing the basis of a large and complex food web (Holguin et al., 2001). Although mangrove ecosystems are rich in organic matter, in general they are nutrient-deficient (Sengupta and Chaudhuri, 1991; Holguin et al., 1992; Alongi et al., 1993; Vazquez et al., 2000). Microorganisms are an important component of the mangrove ecosystem, and there is increasing evidence that microbes are crucial to the biogeochemical productivity of the mangrove ecosystem (Holguin et al., 2001; Thatoi et al., 2013).

In tropical mangroves, bacteria and fungi constitute 91% of the total microbial biomass, whereas algae and protozoa represent only 7 and 2%, respectively (Alongi, 1988). Bacterial communities play an important role in nutrient transformation in mangrove ecosystems (Holguin et al., 2001). Nitrogen fixation [converting gaseous nitrogen (N_2_) to biologically available forms such as ammonia (NH_3_)] by diazotrophs is considered to be the major source of combined nitrogen input in mangrove forest habitats (Kyaruzi et al., 2003). Free-living diazotrophs are widely distributed within the mangrove ecosystem, with high rates of nitrogen fixation detected in association with dead and decomposing leaves, pneumatophores, rhizosphere soil, tree bark, cyanobacterial mats covering the surface of the sediment, and the sediments themselves (Zuberer and Silver, 1978, 1979; Hicks and Silvester, 1985; Holguin et al., 1992, 2001; Lugomela and Bergman, 2002). The high productivity of mangrove ecosystems might be partially attributable to the high rate of biological nitrogen-fixing activity of diazotrophs in sediments and in the rhizosphere of mangrove trees (Holguin et al., 2001).

In recent years, high-throughput sequencing has offered a more comprehensive perspective on microbial communities and has been employed to study the bacterial community associated with mangroves (Dos Santos et al., 2011; Andreote et al., 2012; Gomes et al., 2014; Loganathachetti et al., 2015; Alzubaidy et al., 2016; Wu et al., 2016). The functional diversity and structure of the microbial communities in mangrove wetlands is largely shaped by environmental variables, and each habitat harbors unique microbial functional communities (Bai et al., 2013). Mangrove trees can influence the growth and distribution of microbial communities by enriching the organic carbon pool and changing the redox conditions of the sediments (Holguin et al., 2001). Previous results obtained using acetylene reduction and molecular methods [denaturing gradient gel electrophoresis (DGGE) and terminal restriction fragment length polymorphism (T-RFLP)] showed that the composition and activity of diazotrophs in mangrove ecosystems are strongly influenced by both root–bacterial interactions (Zuberer and Silver, 1978, 1979; Ravikumar et al., 2004; Flores-Mireles et al., 2007) and geochemical parameters (Zhang et al., 2008; Romero et al., 2012). However, the functional *nifH* gene, a marker for diazotrophs, has seldom been examined using high-throughput sequencing in the mangrove rhizosphere (Jing et al., 2014), and little is known comprehensively about the roles that mangrove tree species and environmental variables play in shaping mangove rhizosphere diazotrophic communities. Therefore, it is necessary to examine the diversity, composition, and structure of sediment communities based on both 16S rRNA and *nifH* genes and their links with environmental factors in order to improve our understanding of mangrove ecosystem functioning.

In this study, high-throughput Illumina sequencing was used to investigate microbial communities in the rhizosphere sediments of three species of urban mangrove trees located in Sanya River Mangrove Nature Reserve: *Rhizophora apiculata*, *Avicennia marina*, and *Ceriops tagal*. The aims were to: (i) investigate the diversity and abundance of microbial taxa associated with these three mangrove habitats based on identification of the 16S rRNA and *nifH* genes; (ii) determine the differences in the total microbial composition and the diazotrophic composition among different mangrove species; and (iii) explore the possible relationships between the 16S rRNA and *nifH* gene communities and environmental variables, given the important roles of these microbes in driving biogeochemical cycles in mangrove ecosystems.

## Materials and Methods

### Study Site and Sampling Collection

Three species of mangrove trees were investigated, *R. apiculata*, *A. marina*, and *C. tagal*, which are the dominant species in Sanya River Mangrove Nature Reserve, a typical tropical urban mangrove ecosystem located in the southernmost part of Hainan Island in China. In June 2013, six different sediment cores were collected randomly at each sampling point during low tide. Sediments adjacent to the rhizosphere were collected down to approximately 10 cm. After roots were removed, the sediment was packed on-site into sealed polythene bags. Samples were maintained on ice until transfer to the laboratory. The wet sediments of each core were thoroughly mixed, and subsamples used for nucleic acid extraction were stored at -20°C prior to DNA extraction. Subsamples for environmental parameter analysis were stored at 4°C prior to analysis. Environmental variables including total carbon (TC), total nitrogen (TN), total phosphorus (TP), and available potassium (AK) were measured as described by Bao (1999).

### DNA Extraction, Amplification, Sequencing, and Data Processing

Total community DNA was extracted from 1.0 g of wet sediment using an E.Z.N.A.^®^ Soil DNA Kit (Omega Bio-tek, Norcross, GA, United States). The DNA was then purified with a Promega Wizard DNA Clean-Up System (Madison, WI, United States). DNA concentration was measured by Pico Green using a FLUOstar OPTIMA fluorescence plate reader (BMG Labtech, Jena, Germany).

Two genes were amplified for each sample. To characterize the general 16S rRNA gene community (total microbial composition), the V4 region of the 16S rRNA gene was amplified with the primer pair 515F (5′-GTGCCAGCMGCCGCGGTAA-3′) and 806R (5′-GGACTACHVGGGTWTCTAAT-3′). The diazotrophic microbial community was characterized using the *nifH* gene, which was amplified with primers PolF and PolR (Poly et al., 2001). Both sets of amplicons were modified with Illumina adapter and barcode sequences (Caporaso et al., 2012). Sample libraries were generated from purified PCR products. The Miseq 300 Cycle Kit was used for paired-end sequencing on a Miseq benchtop sequencer (Illumina, San Diego, CA, United States).

The 16S rRNA and *nifH* gene sequences were separated by sample based on their barcodes, permitting up to one mismatch. Quality trimming was done using Btrim (Kong, 2011). Forward and reverse reads were merged into full-length sequences using FLASH (Magoč and Salzberg, 2011). Sequences were removed if they were too short or contained ambiguous bases. For the 16S rRNA gene, operational taxonomic units (OTUs) were classified using UCLUST at the 97% similarity level. Samples were rarefied to 20,000 sequences per sample. OTUs that were only present in a single sample were removed. Taxonomic assignment was conducted using the RDP classifier (release 5.0) (Wang et al., 2013).

For the *nifH* gene, the sequences were analyzed using the FRAMEBOT program (Wang et al., 2013). Sequences having frameshift errors were removed. Error-free sequences were then translated into conceptual protein sequences. The NifH protein sequences were grouped into OTUs using the DOTUR program with a 0.05 sequence distance cutoff (Schloss and Handelsman, 2005; Dang et al., 2009, 2013; Zhou et al., 2016). Samples were rarefied to 12,000 sequences per sample, and singletons were removed. Taxonomic assignment for *nifH* OTUs was carried out by searching representative sequences against reference *nifH* sequences with known taxonomic information. A neighbor-joining (NJ) phylogenetic tree was built by the molecular evolutionary genetics analysis (MEGA6) software (Tamura et al., 2013) for all NifH protein sequences together with selected reference sequences from different diazotrophic groups.

### Statistical Analysis

All analyses were performed using the package vegan in R (R Foundation for Statistical Computing, Vienna, Austria) or our R-based pipeline^1^. Total microbial and diazotrophic species richness and diversity were calculated using the Chao1, Shannon–Wiener (*H*′), and Simpson evenness (*E*) indices. Principal coordinates analysis (PCoA) was used to visualize changes in overall microbial and diazotrophic community structure. Three non-parametric tests [multiple-response permutation procedure (MRPP), permutational multivariate analysis of variance (Adonis), and analysis of similarity (ANOSIM)] were performed based on Bray–Curtis distances to test dissimilarity of 16S rRNA and *nifH* gene communities among mangrove species. Analysis of variance (ANOVA) was performed to identify significant variation in 16S rRNA and *nifH* gene groups among mangrove species. Redundancy analysis (RDA) and the Mantel test were performed to determine the relationships between 16S rRNA and *nifH* gene communities and environmental parameters. All sequences obtained from this study were deposited in the NCBI sequence read archive (SRA) under accession number SRP103888.

## Results

### Environmental Characteristics

The environmental characteristics of the rhizosphere sediment samples from three mangrove species are shown in **Table 1**. All of the measured environmental characteristics including TC, TN, TP, and AK were higher in *R. apiculata* rhizosphere samples than in those from the other two mangrove species. The sediment samples from the *A. marina* rhizosphere had the lowest concentrations of TC, TN, and TP, and had the highest nitrogen to phosphorus ratio (N/P). The sediment samples from the *C. tagal* rhizosphere had the lowest N/P (**Table 1**). The concentrations of TN and AK were higher than observed in our previous investigation (Zhang et al., 2008). The concentration of TP was almost the same as that reported in the Sungei Mandai mangrove ecosystem of Singapore (Jing et al., 2014).

**Table 1:** Environmental characteristics of rhizosphere sediment samples (*n* = 6) from the three mangrove species (expressed as mean value and standard error, *SE*).

	*R. apiculata*	*A. marina*	*C. tagal*
TC (mg/g)	57.50 ± 6.30	19.10 ± 1.88	23.27 ± 4.00
TN (mg/g)	2.83 ± 0.46	1.34 ± 0.18	1.36 ± 0.25
TP (mg/g)	0.35 ± 0.02	0.17 ± 0.02	0.30 ± 0.06
AK (mg/g)	0.61 ± 0.07	0.33 ± 0.04	0.31 ± 0.03
C/N	21.32 ± 1.31	14.60 ± 0.57	17.67 ± 1.08
N/P	7.84 ± 0.79	8.05 ± 0.57	4.63 ± 0.10

### 16S rRNA Gene Composition, Diversity, and Community Structure

**FIGURE 1:**
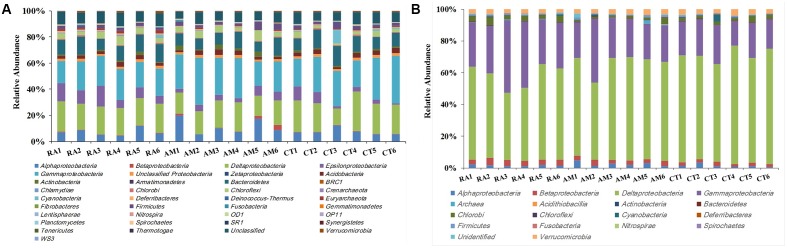
Total 16S rRNA **(A)** and *nifH* **(B)** gene community composition profiles. General 16S rRNA and *nifH* gene taxa were categorized at the phylum level except for the *Proteobacteria*, which were categorized by class. RA, *R. apiculata*. AM, *A. marina*. CT, *C. tagal*.

A total of 406,272 high-quality 16S rRNA gene sequences with lengths of 245–260 bp were obtained. Samples were rarefied to 20,000 sequences per sample. All sequences obtained could be assigned to 24,015 OTUs using UClust (grouped based on 97% similarity). These sequences were classified into 27 bacterial and 2 archaeal phyla (**Figure 1A**). Sequences related to bacteria within the phylum *Proteobacteria* were the most abundant. Within the *Proteobacteria*, *Gammaproteobacteria*, and *Deltaproteobacteria* sequences made up an average of 25 and 21% of the samples, respectively. *Bacteroidetes* was the second most abundant phylum observed in this study, followed by *Chloroflexi*, *Acidobacteria*, and *Firmicutes* (**Figure 1A**).

**FIGURE 2:**
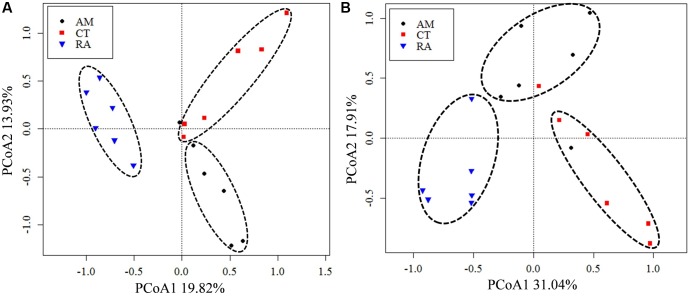
Principal coordinates analysis (PCoA) of total 16S rRNA **(A)** and *nifH* **(B)** gene communities based on high-throughput sequencing data. The percentage of variation explained by each axis is shown. RA, *R. apiculata*. AM, *A. marina*. CT, *C. tagal*.

The 16S rRNA gene communities were highly diverse. The number of OTUs ranged from 8,329 to 14,359 per sample, with *A. marina* harboring the fewest OTUs among the three mangrove species. For individual samples the Shannon–Wiener (*H*′) index ranged from 6.99 to 8.04, and Simpson evenness (*E*) ranged from 0.02 to 0.14. The average Shannon–Wiener (*H*′) of 16S rRNA gene sequence diversity from *R. apiculata* was higher than those from *A. marina* and *C. tagal* (**Table 2**). Three non-parametric tests (MRPP, Adonis, and ANOSIM) were performed using the Bray–Curtis dissimilarity index and consistently showed that microbial communities were significantly different among mangrove species (*P* < 0.02) (**Table 3**). PCoA was also used to compare microbial communities among mangrove species, and the results confirmed that the microbial communities could be divided into three groups corresponding to the mangrove species (**Figure 2A**).

**Table 2:** Diversity indices of 16S rRNA gene and *nifH* sequences from rhizosphere sediments (*n* = 6) of three mangrove species (expressed as mean value and standard error, SE).

	*R. apiculata*		*A. marina*		*C. tagal*	
	16S rRNA	*NifH*	16S rRNA	*NifH*	16S rRNA	*NifH*
	(mean ±*SE*)		(mean ±*SE*)		(mean ±*SE*)	
Chao 1	13,364 ± 328	739 ± 33	11,511 ± 593	682 ± 40	11,549 ± 686	716 ± 29
OTUs	6,622 ± 159	630 ± 26	5,786 ± 254	574 ± 27	5,812 ± 288	610 ± 26
Shannon–Wiener (*H*′)	7.88 ± 0.06	4.90 ± 0.13	7.63 ± 0.08	4.83 ± 0.09	7.57 ± 0.12	4.89 ± 0.09
Simpson *E*	0.10 ± 0.01	0.08 ± 0.01	0.10 ± 0.001	0.09 ± 0.01	0.08 ± 0.01	0.08 ± 0.01

**Table 3:** Non-parametric analyses to test dissimilarity of 16S rRNA and *nifH* gene communities between any two mangrove rhizosphere sediments (RA, *R. apiculata*; AM, *A. marina*; CT, *C. tagal*).

	Adonis		ANOSIM		MRPP	
	Statistic-value	*P*	Statistic-value	*P*	Statistic-value	*P*
*nifH*					
RA vs. AM	4.564	*0.001*	0.691	*0.001*	0.380	*0.002*
RA vs. CT	6.199	*0.001*	0.748	*0.002*	0.379	*0.003*
AM vs. CT	2.719	*0.008*	0.347	*0.014*	0.415	*0.013*
16S rRNA					
RA vs. AM	3.196	*0.001*	0.737	*0.004*	0.530	*0.003*
RA vs. CT	3.295	*0.001*	0.615	*0.002*	0.537	*0.003*
AM vs. CT	1.920	*0.001*	0.246	*0.020*	0.558	*0.001*

*Significant differences (*P* < 0.05) are indicated in italics*.

### Phylogenetic and Taxonomic *nifH* Gene Composition, Diversity, and Community Structure

**FIGURE 3:**
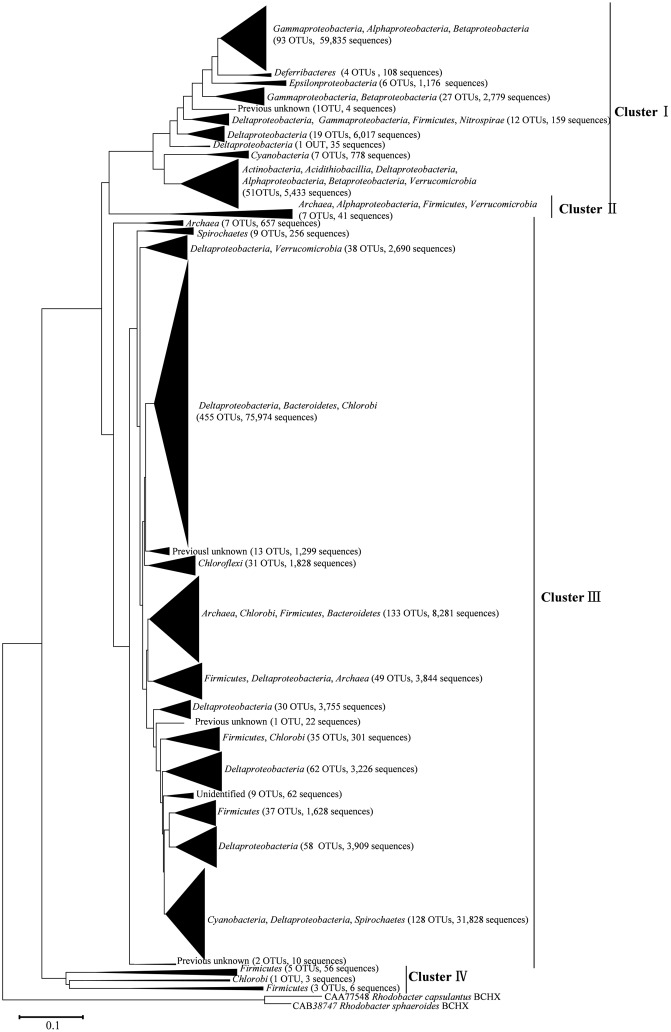
Skeleton phylogenetic tree of the *nifH* sequences reconstructed with the neighbor-joining method from aligned *nifH* sequences as shown in Supplementary Figure S1 in the Supplementary Material. Bootstrap values higher than 50% of 100 resamplings are shown near the corresponding nodes. The chlorophyllide reductase iron protein subunit BchX sequences from *Rhodobacter capsulatus* and *Rhodobacter sphaeroides* were used as an outgroup.

After processing, 216,000 high-quality *nifH* sequences (283–323 bp) were retrieved from the mangrove rhizosphere sediments of *R. apiculata*, *A. marina*, and *C. tagal*. Samples were rarefied to 12,000 sequences per sample. All sequences obtained could be assigned to 1,334 OTUs at the 95% protein sequence similarity level (Dang et al., 2009, 2013; Zhou et al., 2016). The 1,334 unique *nifH* protein sequences shared 51–100% sequence identity with the top-match sequences obtained from GenBank. Among these, 591 unique protein sequences (comprising up to 82.82% of total sequences) shared quite high sequence identity (>90%) with *nifH* sequences of known bacteria or archaea, such as *Deltaproteobacteria*, *Acidithiobacillia*, *Alphaproteobacteria*, *Gammaproteobacteria*, *Bacteroidetes*, *Cyanobacteria*, *Chlorobi*, *Deferribacteres*, *Epsilonproteobacteria*, *Firmicutes*, *Spirochaetes*, *Verrucomicrobia*, and *Euryarchaeota*. Phylogenetic types of *nifH* genes were defined according to Zehr et al. (2003). The deduced *nifH* sequences were affiliated with four major groups in the reconstructed *nifH* phylogenetic tree (**Figure 3** and Supplementary Figure S1). The *nifH* community was dominated by sequences belonging to Cluster I and Cluster III *nifH* clades, which accounted for 35.33 and 64.61% of the total captured sequences and 16.56 and 82.23% of the total OTUs, respectively. Only seven OTUs accounting for 0.52% of total sequences were found to belong to Cluster II, and nine OTUs accounting for 0.67% of total sequences were found in Cluster IV (**Figure 3** and Supplementary Figure S1). *R. apiculata* harbored significantly more *nifH* sequences belonging to Cluster I and fewer *nifH* sequences belonging to Cluster III than did *A. marina* and *C. tagal* (Supplementary Tables S1, S2).

Family or higher taxonomic information was then assigned to the 1,334 *nifH* OTUs, according to their nearest taxonomic matches, for further analyses. All *nifH* sequences were classified into 1 archaeal and 11 bacterial phyla (**Figure 1B**). Sequences related to diazotrophs within the phylum *Proteobacteria* were the most abundant. Within the *Proteobacteria*, *Deltaproteobacteria*-related sequences were the most abundant group, making up an average of 60.59% of diazotrophic sequences. *Gammaproteobacteria* were the second major diazotrophic group, making up an average of 26.07% diazotrophic sequences (**Figure 1B**). The number of diazotrophic OTUs ranged from 490 to 715 across all the samples, with *C. tagal* harboring the highest number of OTUs among the three mangrove species. The Shannon–Wiener (*H*′) index ranged from 4.51 to 5.21, and Simpson evenness (*E*) ranged from 0.05 to 0.12 for individual samples. The average Shannon–Wiener (*H*′) index of diazotrophic diversity from *A. marina* was relatively lower than those of *A. marina* and *C. tagal* (**Table 2**). Dissimilarity tests showed that the diazotrophic communities were significantly different among mangrove species (*P* < 0.01) (**Table 3**). PCoA also showed significant variations in the diazotrophic communities from different mangrove species (**Figure 2B**).

### Comparison of 16S rRNA and *nifH* Gene Composition among Samples from the Three Mangrove Species

**FIGURE 4:**
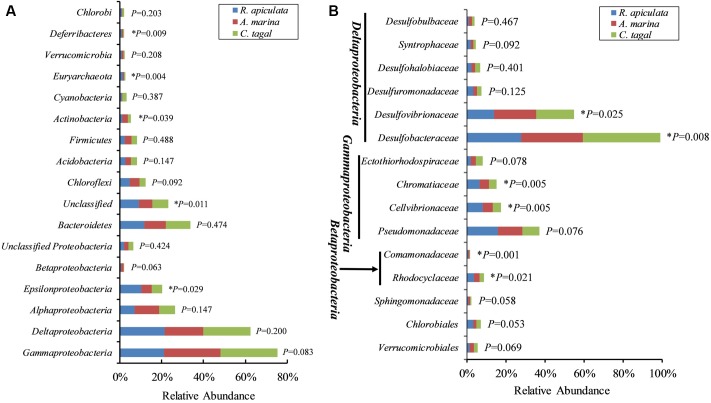
Relative abundance of the most abundant 16S rRNA and *nifH* gene groups. **(A)** The dominant 16S rRNA gene groups were categorized at the phylum level, except for the *Proteobacteria*, which were categorized by class. **(B)** The dominant *nifH* gene groups were classified to the family level. Asterisks (^∗^) represent significant differences among sediments of the three mangrove species (*P* < 0.05).

The effect of mangrove tree species on 16S rRNA and *nifH* gene distribution was further investigated using the most dominant 16S rRNA (*n* = 17) and *nifH* (*n* = 15) gene groups (**Figure 4**). The results showed that most of the dominant microbial groups had no significant differences in relative abundance in the communities from the three mangrove species. Of these dominant 16S rRNA gene groups, *Epsilonproteobacteria*, *Deferribacteres*, and *Euryarchaeota* were highly abundant in rhizosphere sediment from *R. apiculata*, while *Actinobacteria* dominated in sediment from *A. marina*. However, *Gammaproteobacteria* and *Deltaproteobacteria*, the two most dominant bacterial groups across all of the mangrove species, were almost invariable (**Figure 4A**).

In contrast to the total microbial pattern at the phylum level, most of the dominant diazotrophic groups were significantly different among the three mangrove species (*P* < 0.05). The abundance of the two most dominant diazotrophic groups, *Desulfobacteraceae* and *Desulfovibrionaceae*, belonging to the *Deltaproteobacteria*, was significantly different among the mangrove species (*P* < 0.05), and these groups accounted for an average of 33.02 and 18.29% of total diazotrophic sequences, respectively. *Desulfobacteraceae* were highly abundant in rhizosphere sediment from *C. tagal*, while *Desulfovibrionaceae* dominated in *A. marina*. The *Pseudomonadaceae* of the *Gammaproteobacteria*, the third most dominant diazotrophic group, were almost invariable, accounting for an average of 12.38% of total diazotrophic sequences. Two dominant *Betaproteobacteria* diazotrophic groups, *Rhodocyclaceae* and *Comamonadaceae*, were relatively highly abundant in rhizosphere sediment from *R. apiculata*. The diazotrophic groups belonging to the *Verrucomicrobia* and *Firmicutes* were almost invariable (**Figure 4B**).

### Relationship between 16S rRNA and *nifH* Gene Communities and Environmental Factors

**FIGURE 5:**
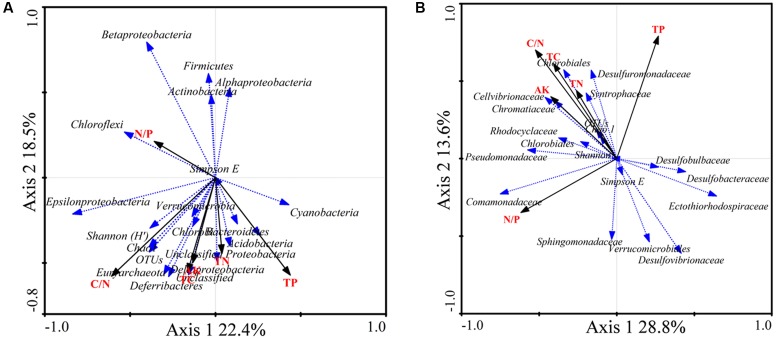
Redundancy analysis (RDA) ordination biplot showing the relationships between environmental variables and total 16S rRNA **(A)** and *nifH* **(B)** gene groups. Only the most abundant groups are shown in the biplot.

Redundancy analysis was performed to explore the relationship between 16S rRNA and *nifH* gene community structures with different environmental characteristics. The results showed that the composition of diazotrophic communities was significantly correlated with all investigated environmental factors (Monte Carlo test, *P* < 0.05; **Table 4**) except the concentration of TN. The total 16S rRNA gene community did not significantly correlate with TN or N/P ratios of mangrove sediments (**Table 4**). However, the diversity of both 16S rRNA and *nifH* genes did not significantly correlate with investigated environmental factors (**Figure 5**).

**Table 4:** Monte Carlo permutation test of relationship between environmental attributes and 16S rRNA and *nifH* gene high-throughput sequencing data.

	16S rRNA		*nifH*	
	*R*^2^	*P*	*R*^2^	*P*
TP	0.341	*0.037*	0.534	*0.004*
TN	0.278	0.104	0.293	0.073
TC	0.377	*0.024*	0.509	*0.012*
AK	0.360	*0.024*	0.404	*0.023*
C/N	0.320	*0.045*	0.559	*0.004*
N/P	0.193	0.196	0.403	*0.014*

*Significant differences (*P* < 0.05) are indicated in italics*.

The RDA biplot showed that the abundance of sequences from the 16S rRNA groups *Chloroflexi*, *Betaproteobacteria*, *Alphaproteobacteria*, *Firmicutes*, and *Actinobacteria* was positively correlated with the N/P ratio. *Gammaproteobacteria*, *Cyanobacteria*, *Acidobacteria*, and *Deltaproteobacteria* were positively correlated with TP. In addition, *Epsilonproteobacteria*, *Deferribacteres*, and *Euryarchaeota* were positively correlated with the C/N ratio, TC, and AK (**Figure 5A**).

The diazotrophic groups *Deltaproteobacteria*, *Desulfobulbaceae*, and *Desulfuromonadaceae* were positively correlated with TP. The diazotrophic gammaproteobacterial families *Pseudomonadaceae*, *Cellvibrionaceae*, and *Chromatiaceae* were positively correlated with the C/N ratio, TC, TN, and AK. The diazotrophic groups *Deltaproteobacteria*, *Desulfovibrio*, and *Desulfobacca* were negatively correlated with the C/N ratio, TC, TN, and AK. Diazotrophic *Deltaproteobacteria* and *Desulfobacter* were positively correlated with the N/P ratio (**Figure 5B**).

## Discussion

Although high-throughput sequencing approaches are now commonly applied to investigate bacterial community structures in mangroves (Dos Santos et al., 2011; Andreote et al., 2012; Gomes et al., 2014; Loganathachetti et al., 2015; Alzubaidy et al., 2016; Wu et al., 2016), they have not been widely used to target nitrogen-fixing functional genes (*nifH*) to explore diazotrophic communities in urban mangrove rhizospheres (Jing et al., 2014). Compared with previous high-throughput sequencing studies on diazotrophic communities from the mangroves along the coastline of Singapore (Jing et al., 2014), we found a much greater diversity of bacteria and archaea having the potential to fix nitrogen from the three rhizosphere sediments from *R. apiculata*, *A. marina*, and *C. tagal*, which might be attributable to differences in mangrove species, environmental conditions, and primers used for analysis. The primers we used have a very broad coverage for both bacterial and archaeal *nifH* genes, and have been widely used in various environmental studies (Bernard et al., 2014; Hoppe et al., 2014; Keshri et al., 2015; Zhang et al., 2015; Newell et al., 2016; Penton et al., 2016; Tu et al., 2016; Rodrigues et al., 2017).

### 16S rRNA and *nifH* Gene Community Structures from Rhizosphere Sediments of *R. apiculata*, *A. marina*, and *C. tagal*

Previous studies have reported *Deltaproteobacteria* and *Gammaproteobacteria* as the dominant bacterial groups in rhizosphere sediments from many mangrove species (Gomes et al., 2014; Loganathachetti et al., 2015; Alzubaidy et al., 2016; Wu et al., 2016). In this present study, *Deltaproteobacteria* and *Gammaproteobacteria* were the dominant groups of both total bacterial and diazotrophic communities from all three mangrove species studied. These two groups constituted an average of 45.87 and 86.66% of total 16S rRNA and *nifH* gene communities, respectively (**Figure 1**), suggesting that these groups play an important functional role in the anaerobic conditions of the mangrove rhizosphere. Furthermore, there was a high relative abundance of diazotrophic groups from the *Deltaproteobacteria* in this study, such as *Desulfobacteraceae*, *Desulfovibrionaceae*, and *Desulfuromonadaceae*, belonging to the sulfate-reducing bacteria, which are known to play key roles in sedimentary cycling of N, C, and S (Lyimo et al., 2002; Varon-Lopez et al., 2014; Romero et al., 2015). Sulfate-reducing bacteria were also reported to be prevalent in pristine, anthropogenic, and oil-contaminated mangrove sediments (Santos et al., 2011; Jing et al., 2014; Wu et al., 2016), which suggests that sulfate-reducing bacteria may contribute substantially to both nitrogen fixation and sulfate reduction in the mangrove rhizosphere. Furthermore, our findings reinforce the prominence of sulfate-reducing bacteria as the main diazotrophic group in mangrove samples. In addition, diazotrophs from the *Gammaproteobacteria* were reported to be widespread in tropical and subtropical oceans (Bird et al., 2005; Jing et al., 2014). The family *Pseudomonadaceae* is a common constituent of mangrove rhizosphere diazotrophs (Zhang et al., 2008; Liu et al., 2012; Jing et al., 2014), and it was detected as the dominant group of the class *Gammaproteobacteria* in this study.

In addition, *Bacteroidetes* and *Firmicutes*, including a high abundance of sulfate reducers and methanogens, were dominant in sediments of *A. marina* (Alzubaidy et al., 2016). *Bacteroidetes* are very frequent in tidal mudflats or near-shore sediments, and an increased abundance of *Bacteroidetes* in the rhizospheres of mangroves has been noted for *Rhizophora mangle*, *Avicennia schaueriana*, and *Laguncularia racemosa* located in Guanabara Bay (Rio de Janeiro, Brazil) (Gomes et al., 2010). *Actinobacteria*, including mostly soil-borne microbes, were reported to be enriched in mangrove sediments in the Red Sea (Alzubaidy et al., 2016). *Chloroflexi* was the second most dominant phylum in three mangrove species in Beilun Estuary (Wu et al., 2016). Consistent with previous reports, bacteria from the phyla *Bacteroidetes*, *Chloroflexi*, *Acidobacteria*, and *Firmicutes* were also widespread in the rhizosphere sediments of *R. apiculata*, *A. marina*, and *C. tagal*.

### Influence of Mangrove Species and Environmental Factors on 16S rRNA and *nifH* Gene Composition from Rhizosphere Sediments

Several factors shaping mangrove rhizosphere microbial communities have been proposed (Alzubaidy et al., 2016). Mangrove tree species and geochemical parameters were widely reported to be influential (Flores-Mireles et al., 2007; Zhang et al., 2008; Gomes et al., 2010, 2014; Romero et al., 2012; Jing et al., 2014; Wu et al., 2016). Mangrove roots have been suggested to be able to impose a selective force on the mangrove rhizosphere microbial communities. This phenomenon appeared to be plant species-specific (Gomes et al., 2014), resulting in the mangrove tree species playing an important role in shaping the rhizosphere microbial communities (Gomes et al., 2010, 2014; Pires et al., 2012; Wu et al., 2016). Mangrove root exudates may not only stimulate microbial respiration and create suboxic and anoxic microenvironments to facilitate nitrogen fixation, but also directly stimulate nitrogen fixation by providing metabolizable organic matter as carbon and energy sources to the diazotrophs (Dang and Lovell, 2016). The significant influence of the mangrove tree species on the rhizosphere microbial community was further confirmed in this study. Three non-parametric tests based on the Bray–Curtis distance matrix showed that both the total 16S rRNA and the *nifH* gene communities were significantly different among mangrove species (*P* < 0.02) (**Table 2**). The PCoA results also confirmed that the 16S rRNA and *nifH* gene communities could be divided into three groups corresponding to the respective mangrove species (**Figure 2**). The differences in 16S rRNA gene community composition from the three investigated mangrove species showed that the dominant 16S rRNA gene groups *Epsilonproteobacteria*, *Actinobacteria*, *Deferribacteres*, and *Euryarchaeota* were significantly different in abundance in association with different mangrove species (**Figure 4A**). Our results are consistent with the idea that root exudates select for specific groups at both the taxonomic level and the functional level (Alzubaidy et al., 2016). Most of the dominant diazotrophic groups were significantly different among mangrove species (*P* < 0.05). The possible reason for plant species-specific diazotrophic communities is that mangroves of different species and under different physiological conditions may secrete different types of organic matter, which selects different diazotrophic species to be functional (Dang and Lovell, 2016).

The importance of geochemical parameters in structuring 16S rRNA and *nifH* gene communities has been previously shown in mangrove ecosystems (Flores-Mireles et al., 2007; Zhang et al., 2008; Romero et al., 2012; Jing et al., 2014). Our results showed that both total 16S rRNA and *nifH* gene communities significantly correlated with most of the investigated environmental factors (**Table 4**). Mangrove root exudates provide a valuable source of carbon, while microorganisms that colonize the rhizosphere help plants acquire phosphorus and potassium; enhance nitrogen uptake; or even help the plants to cope with infection, toxic compounds, and other sources of stress (Singh et al., 2004; Kristensen et al., 2008). In this study, the fact that the highest 16S rRNA and *nifH* gene diversity was found in samples from the *R. apiculata* rhizosphere is not surprising, as these samples have a relatively high TC concentration. It has been reported that long-term fertilization with nitrogen and phosphorus not only affects the community structure and activity of diazotrophs, but also potentially plant–microbe interactions (Romero et al., 2012, 2015). Diazotroph diversity is reduced or completely eliminated by high levels of ammonia caused by anthropogenic activities, while relatively high phosphorus concentrations provide favorable conditions for nitrogen fixation (Jing et al., 2014). The abundance of the dominant diazotroph *Desulfatibacillum* in this study was positively correlated with TP, but negatively correlated with the N/P ratio. Since plants require potassium for numerous physiological processes such as growth and development, and protein synthesis, there is interest in bacteria such as *Pseudomonas*, which are capable of solubilizing potassium to an accessible form in the soil (Alzubaidy et al., 2016). In the present study, *Pseudomonadaceae* abundance was positively correlated with the concentration of AK. Among the family *Pseudomonadaceae*, most protein sequences shared quite high sequence identity (>90%) with those of the known bacteria *Pseudomonas stutzeri*, which further hints at the important role that diazotrophic *Pseudomonas* play in potassium cycling. It is notable that more than half of the variability (>50%) was unexplained by the host species or environmental variables investigated in this study, for both the 16S rRNA and *nifH* gene communities. Other unknown factors such as redox, pH, oxygen content, organic matter content, and salinity may be important in influencing the diversity, abundance, structure, and spatial distribution of the sediment *nifH* gene communities (Dang et al., 2009, 2013; Zhou et al., 2016).

## Conclusion

Our study comprehensively characterized the diversity and structure of 16S rRNA and *nifH* gene communities in the rhizosphere of three mangrove species. Both the mangrove species and various environmental variables played important roles in shaping these communities. Most of the dominant diazotrophs were significantly different among mangrove species. However, our study was based on DNA abundance, so activity of nitrogenase cannot be confirmed. Therefore, expression-based studies such as mRNA-based microarray hybridization and metagenomic studies, together with *in situ* nitrogen-fixing measurements, are required to elucidate the role that diazotrophs play in the mangrove rhizosphere.

## Author Contributions

YZ, JL, and JD conceived the research. YZ, QY, and JL performed the experiments. YZ wrote the manuscript. JVN and JZ edited the manuscript. QY, JL, and ZS contributed sampling or data analysis pipelines. All authors reviewed and approved the manuscript.

## Conflict of Interest Statement

The authors declare that the research was conducted in the absence of any commercial or financial relationships that could be construed as a potential conflict of interest.

## Footnotes

1 http://www.ou.edu/ieg/tools/data-analysis-pipeline.html
