# Supplementary material for: Diversity and Structure of Diazotrophic Communities in Mangrove Rhizosphere, Revealed by High-Throughput Sequencing
Source: Front Microbiol. 2017 Oct 18;8:2032. doi: 10.3389/fmicb.2017.02032 (PMC5651520; doi:10.3389/fmicb.2017.02032)
Supplement: Supplementary file 1 [file Presentation_1.PPTX]

## Slide 1
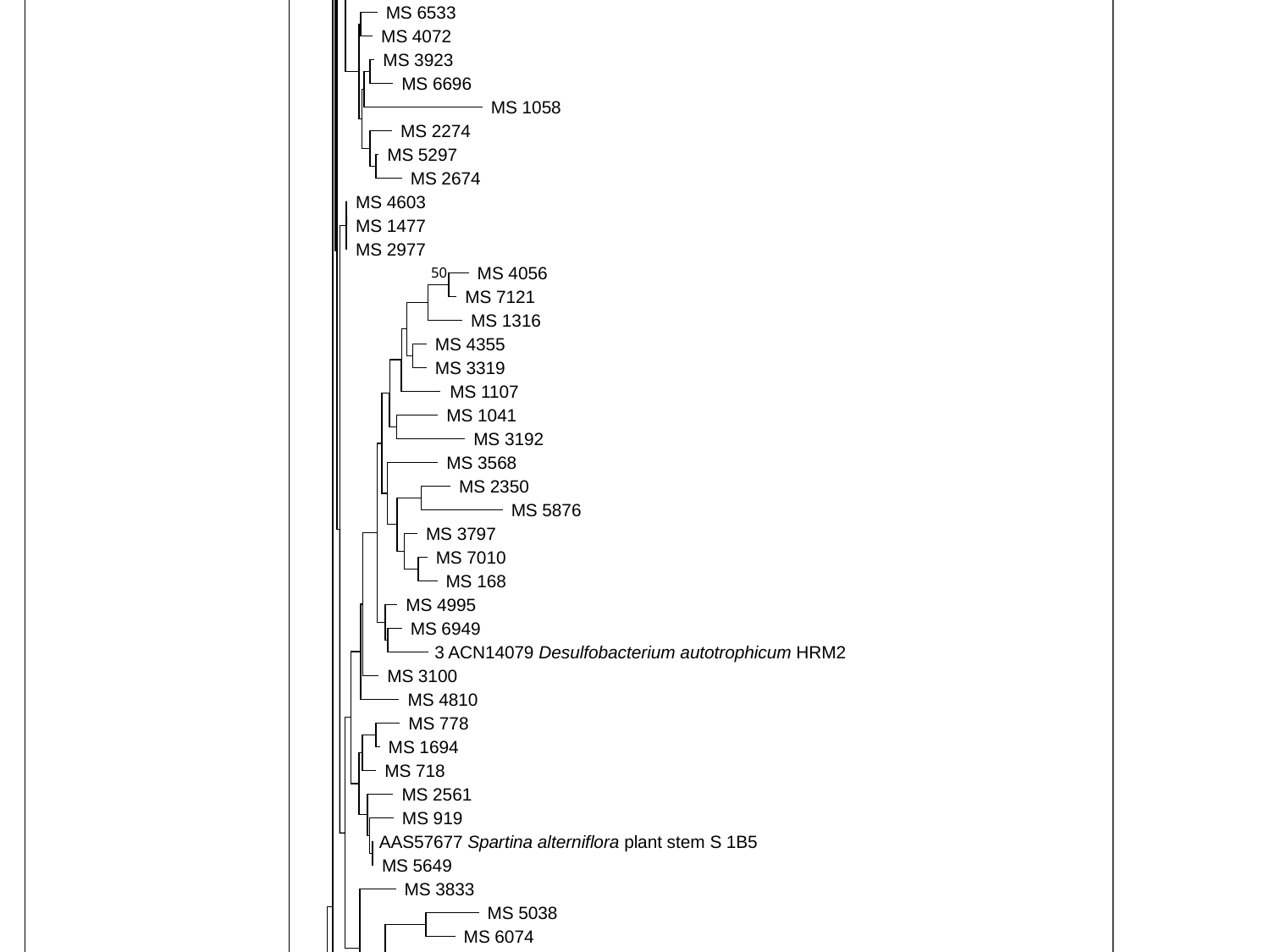

Supplementary Material
Diversity and structure of microbial diazotrophic communities in a mangrove rhizosphere as revealed by high throughput sequencing
Yanying Zhang, Qingsong Yang, Juan Ling, Joy D. Van Nostrand, Zhou Shi, Jizhong Zhou, Junde Dong*
* Correspondence: Junde Dong: dongjd@scsio.ac.cn
Supplementary Figures
 MS 6480
 MS 5282
 MS 3566
 AFWU01000019 Marichromatium purpuratum 984
 MS 6428
 MS 1581
 MS 5758
 MS 1323
 MS 5885
 CP000089 Dechloromonas aromatica RCB
 MS 5160
 MS 4947
 MS 814
 MS 697
 MS 4528
 MS 6899
 MS 3726
 MS 3688
 MS 6104
 MS 1212
 AEGW01000005 Methylobacter tundripaludum SV96
 MS 386
 MS 3259
 MS 2336
 MS 4186
 MS 882
 MS 6164
 MS 2986
 MS 3134
 MS 4416
 MS 3452
 MS 5840
 MS 2646
 AGFC01000057 Thiocystis violascens DSM 198
 MS 6638
 MS 5882
 AGFD01000008 Thiorhodospira sibirica ATCC 700588
 MS 6402
 MS 6800
 CP000471 Magnetococcus sp. MC-1
 MS 1699
 MS 806
 MS 1154
 MS 6396
 MS 1816
 MS 1740
 MS 95
 MS 2266
 MS 1821
 CP000304 Pseudomonas stutzeri A1501
 MS 1087
 MS 5645
 MS 4658
 MS 6226
 MS 4730
 MS 1233
 MS 315
 MS 6655
 ACQQ01000002 Allochromatium vinosum DSM 180
 MS 1485
 MS 4325
 MS 2756
 MS 2110
 MS 4432
 CP000964 Klebsiella pneumoniae 342
 MS 6720
 MS 6750
 MS 6867
 CP001614 Teredinibacter turnerae T7901
Cluster I
 MS 4446
 AH000924 Rhizobium sp.
 MS 3469
 ADNR01000001 Burkholderia sp. Ch1-1
 MS 5306
 MS 2388
 AB094963 Bradyrhizobium elkanii USDA 76
 MS 2697
 MS 3549
 M16709 Rhizobium sp. 1
 MS 3640
 MS 5825
 CP000529 Polaromonas naphthalenivorans CJ2
 MS 3567
 CP000781 Xanthobacter autotrophicus Py2
 FQ859181 Hyphomicrobium sp. MC1
 MS 4724
 AEWJ01000022 Novosphingobium nitrogenifigens DSM 19370
 MS 268
 MS 3964
 MS 5703
 CP000975 Methylacidiphilum infernorum V4
 MS 3065
 MS 4569
 CP001013 Leptothrix cholodnii SP-6
 MS 4426
 AEVM01000015 Methylocystis sp. ATCC 49242
 CP001016 Beijerinckia indica subsp. indica ATCC 9039
 MS 2911
 2 CAA32055 Methanothermococcus thermolithotrophicus
 2 ABK78681 Hyperthermophilic methanogen FS406-22
 MS 3998
 MS 7015
 CP001096 Rhodopseudomonas palustris TIE-1
 2 ACF14285 Chloroherpeton thalassium ATCC 35110
 2 CAA39552 Methanosarcina barkeri
 MS 3637
Cluster II
 CP002547 Syntrophobotulus glycolicus DSM 8271
 2 ADU27821 Ethanoligenens harbinense YUAN-3
 MS 2643
 ABEA02000149 Opitutaceae bacterium TAV2
 MS 4240
 2 P09553 Clostridium pasteurianum
 MS 5973
 MS 6410
 MS 4084
 MS 5857
 CP002565 Methanosaeta concilii GP-6
 MS 429
 MS 5682
 MS 3659
 MS 2516
 ADJQ01000025 Ethanolins harbinense YUAN-3
 MS 483
 MS 3342
 MS 6186
 MS 4019
 MS 6972
 MS 3908
 CP001698 Spirochaeta thermophila DSM 6192
 MS 1814
 CP001843 Treponema primitia ZAS-2
 MS 1439
71
69
72
82
63
94
51
89
95
65
60
95
66
88
77
76
51
100
98
55
96
79
100
75
50
58
57
73
53
92
94
98
99
100
95
97
53
70
85
82
56
97
79
100
94
93
83
83
69
79
72
79
100
94
100
100
92
56
89
53
94
100
98
98
93
91
99
99
92
96
77
83
70
98
96
92
97
87
97
78
68
70
96
84
67
92
86
99
100
73
100
90
79
70
54
100
76
99
98
97
76
96
93
89
99
99
94
99
99
97
88
93
100
100
100
60
96
97
97
100
100
94
85
52
95
89
74
68
90
100
98
71
56
65
71
100
100
 ACEP01000004 Eubacterium hallii DSM 3353
 MS 522
100
 CP002770 Desulfotomaculum kuznetsovii DSM 6115
100
 MS 5735
 4A AEF84422 Treponema primitia ZAS-2
Cluster IV
 4A EFF64704 Turicibacter sanguinis PC909
 CP001097 Chlorobium limicola DSM 245
100
 MS 4289
 4A ACL15489 Methanosphaerula palustris E1-9c
 ABVR01000032 Coprococcus comes ATCC 27758
100
 MS 6388
 FP929038 Coprococcus catus GD
100
65
 MS 2328
 AEDJ01000005 Ruminococcus albus 7
54
 MS 6692
100
 CAA77548 Rhodobacter capsulantus BCHX
 CAB38747 Rhodobacter sphaeroides BCHX
100
 MS 4611
 MS 6421
 MS 6688
 MS 4299
 MS 1229
 MS 6118
 AB189453 Pseudomonas azotifigens
 MS 5727
 CP001157 Azotobacter vinelandii DJ
 MS 4770
 CP002738 Methylomonas methanica MC09
 MS 3504
 MS 618
 MS 4087
 MS 4869
 MS 644
 MS 2429
 MS 928
 MS 5017
 AFWS01000002 Thiorhodovibrio sp. 970
 MS 5736
 MS 1359
 AFWV01000003 Thiocapsa marina 5811
 MS 3004
 MS 5351
 MS 4134
 MS 2342
 MS 4003
 MS 5723
 MS 3483
 MS 1822
 MS 5621
 M20568 Azotobacter vinelandii
 MS 4350
 MS 3817
 MS 3473
 MS 3233
 MS 4163
 MS 5530
 MS 191
 MS 6160
 MS 4725
 CP002347 Calditerrivibrio nitroreducens DSM 19672
 MS 4912
 ABTN01000004 Denitrovibrio acetiphilus DSM 12809
 MS 6671
 CP002355 Sulfuricurvum kujiense DSM 16994
 MS 1666
 MS 1013
 BX571660 Wolinella succinos
 MS 1247
 MS 4313
 MS 5603
 MS 748
 AP012048 Arcobacter sp. L
 MS 4895
 MS 6582
 MS 6988
 MS 3478
 AF200742 Azoarcus sp. BH72
 MS 5315
 AB189641 Halorhodospira halophila
 MS 2483
 MS 2818
 MS 1118
 MS 2604
 MS 4208
 MS 4747
 MS 710
 MS 2431
 MS 1624
 MS 6425
 MS 4873
 MS 4976
 MS 5308
 MS 6089
 MS 4638
 MS 4879
 MS 4006
 MS 4054
 MS 6062
 MS 4852
 MS 3409
 CP001965 Sideroxydans lithotrophicus ES-1
 MS 2458
 MS 3128
 AB196525 Heliobacterium chlorum nifI1
 MS 2517
 2 BAE86060 Desulfitobacterium hafniense Y51
 MS 5558
 CP000482 Pelobacter propionicus DSM 2379
 MS 6177
 CP001147 Thermodesulfovibrio yellowstonii DSM 11347
 MS 5353
 CM001230 Brenneria sp. EniD312
 AGAF01000125 Desulfosporosinus sp. OT
 MS 5171
 MS 6228
 MS 2362
 MS 4373
 CP003108 Desulfosporosinus orientis DSM 765
 MS 1627
 MS 5349
 MS 5756
 MS 5359
 CP001089 Geobacter lovleyi SZ
 MS 1868
 CP001124 Geobacter bemidjiensis Bem
 MS 1267
 CP000698 Geobacter uraniireducens Rf4
 MS 5818
 MS 6779
 MS 4983
 MS 4687
 MS 2258
 CP001131 Anaeromyxobacter sp. K
 MS 5586
 MS 5785
 MS 2878
 CP000769 Anaeromyxobacter sp. Fw109-5
 MS 2520
 CP000148 Geobacter metallireducens GS-15
 MS 2682
 CP000142 Pelobacter carbinolicus DSM 2380
 MS 4574
 MS 3482
 MS 3731
 MS 4044
 MS 3195
 MS 6822
 1 EAT15955 Desulfuromonas acetoxidans DSM 684
 AAEW02000007 Desulfuromonas acetoxidans DSM 684
 MS 3776
 1 ALC17315 Desulfuromonas soudanensis
 MS 3946
 U49859 Anabaena variabilis ATCC 29413
 MS 3701
 MS 70
 D00666 Leptolyngbya boryana
 MS 411
 FJ170277 Candidatus Atelocyanobacterium thalassa isolate ALOHA
 MS 473
 MS 993
 AB293988 Gloeothece sp. KO68DGA
 DS989904 Synechococcus sp. PCC 7335
 MS 3542
 MS 1308
 CP000393 Trichodesmium erythraeum IMS101
 MS 6448
 FQ311868 Azospirillum lipoferum 4B
 MS 6589
 AP010946 Azospirillum sp. B510
 MS 3124
 AFJD01000006 Acidithiobacillus ferrivorans SS3
 MS 5488
 MS 3517
 CP001192 Rhizobium leguminosarum bv. trifolii WSM2304
 MS 2715
 MS 1550
 M10587 Rhizobium phaseoli
 MS 4832
 MS 3436
 ADGX01000041 Frankia sp. EUN1f
 MS 6983
 MS 6938
 AEWG01000097 Rubrivivax benzoatilyticus JA297
 MS 5302
 MS 2861
 MS 727
 CP000249 Frankia sp. CcI3
 MS 363
 ADGT01000052 Frankia symbiont of Datisca glomerata
 CT573213 Frankia alni str ACN14A
 MS 6182
 CP000943 Methylobacterium sp. 4-46
 MS 5693
 MS 2511
 CP001280 Methylocella silvestris BL2
 MS 6670
 ACYY01000009 Rhodobacter sp. SW2
 MS 4060
 NC2678 Mesorhizobium loti MAFF303099
 MS 3153
 JN021929 Rhizobium sp. CCGE 501
 MS 6850
 MS 6797
 MS 6788
 MS 6181
 CP000463 Rhodopseudomonas palustris BisA53
 MS 2886
 MS 5383
 AF194084 Unidentified bacterium
 MS 4439
 ACQU01000014 Zymomonas mobilis subsp. mobilis ATCC 10988
 MS 5303
 MS 252
 MS 6547
 AM889285 Gluconacetobacter diazotrophicus PAl 5
 MS 1110
 MS 669
 CP001132 Acidithiobacillus ferrooxidans ATCC 53993
 MS 5169
69
Cluster III
 MS 5098
 WP 071544382 Desulfovibrio dechloracetivorans
 MS 7003
 MS 3808
 MS 4678
 MS 5745
 3 ADH87119 Desulfurivibrio alkaliphilus AHT 2
 WP 027723058 Desulfovibrio zosterae
 MS 6127
 MS 910
 MS 6147
 AHN50620 MD soil MDE amb 29e7
 MS 4022
 MS 224
 MS 5135
 MS 1879
 MS 5878
 MS 2124
 MS 1458
 MS 5041
 MS 3241
 MS 220
 MS 4266
 MS 3069
 MS 3622
 MS 1192
 MS 6294
 MS 2368
 ABTP01000003 Desulfomicrobium baculatum DSM 4028
 MS 6873
 MS 307
 MS 4188
 MS 2708
 MS 2416
 MS 4676
 MS 3157
 MS 5470
 MS 5442
 MS 2508
 MS 2044
 MS 4921
 CM001077 Desulfovibrio sp. ND132
 MS 4245
 MS 5434
 MS 6284
 MS 62
 MS 3506
 ACX68565 mangrove Rhizophora mangle sediment RN17
 MS 2908
 MS 3044
 OGR04953 Deltaproteobacteria bacterium RIFOXYD12 FULL 53 23
 AEM06411 High Canadian Arctic Mackenzie River MCK.River 61
 MS 3067
 MS 1008
 ACBZ01000044 Blautia hydrogenotrophica DSM 10507
 MS 7031
 MS 2098
 ABEZ02000019 Clostridium bartlettii DSM 16795 C
 CP001841 Treponema azotonutricium ZAS-9
 MS 1601
 MS 7017
 MS 1953
 MS 375
 CP000528 Desulfovibrio vulgaris DP4
 MS 1656
 MS 6762
 MS 1463
 MS 6834
 MS 3000
 MS 589
 MS 2177
 MS 6742
 MS 5117
 MS 6791
 MS 4525
 MS 2218
 MS 4713
 MS 6749
 MS 1600
 CP001197 Desulfovibrio vulgaris str. Miyazaki F
 MS 5013
 MS 4619
 MS 4363
 MS 5115
 MS 1955
 MS 2990
 MS 6171
 MS 7023
 MS 4659
 MS 7128
 MS 3320
 MS 4035
 MS 5695
 MS 293
 MS 3668
 MS 6129
 MS 2527
 MS 1594
 MS 3683
 MS 1352
 MS 1217
 MS 135
 MS 3824
 CP001998 Coraliomargarita akajimensis DSM 45221
 MS 4718
 MS 1588
 MS 3032
 MS 4116
 AHN50707 MD soil MDE amb 35a2
 MS 3078
 MS 6399
 MS 3613
 MS 4094
 MS 3891
 MS 1462
 MS 2550
 MS 2876
 MS 4113
 MS 5119
 MS 6838
 MS 1280
 ADFE01000001 Desulfovibrio sp. FW1012B
 MS 5945
 MS 5328
 MS 4899
68
66
95
54
51
52
57
60
 MS 3470
 MS 1917
 MS 713
 MS 868
 MS 7107
 MS 3893
 MS 3279
 MS 1775
 MS 4142
 MS 5972
 MS 4591
 MS 658
 MS 5545
 MS 4907
 MS 1145
 MS 4797
 MS 3278
 MS 5338
 MS 6728
 MS 2856
 MS 6736
 MS 6301
 MS 6418
 ADLV01000010 Dysgonomonas gadei ATCC BAA-286
 MS 2372
 MS 5505
 MS 3337
 MS 5060
 ABB21307 salt pond microbial mat nif1004220L32
 MS 6569
 MS 5231
 MS 5801
 MS 6933
 MS 4466
 MS 4900
 MS 6994
 MS 228
 MS 5162
 MS 425
 MS 6132
 AHG52700 hand-picked Trichodesmium nifH46
 MS 2306
 MS 5332
 MS 6214
 MS 3197
 MS 2924
 MS 5812
 MS 5406
 MS 6206
 MS 5305
 MS 4244
 MS 1468
 MS 3562
 MS 693
 MS 5970
 MS 1729
 MS 4255
 MS 6036
 MS 4578
 MS 4207
 MS 6676
 MS 3112
 MS 6065
 MS 3512
 MS 3171
 MS 4493
 MS 3913
 MS 4257
59
86
89
54
51
 MS 855
 MS 3187
 MS 4086
 MS 3971
 MS 6161
 MS 2969
 MS 3082
 MS 5385
 MS 4844
 MS 216
 AECZ01000006 Desulfovibrio fructosovorans JJ
 MS 441
 MS 3477
 MS 2254
 MS 235
 MS 4263
 MS 4758
 MS 3073
 MS 2882
 MS 2345
 MS 6311
 MS 2441
 MS 6737
 MS 2649
 MS 732
 MS 175
 MS 2334
 MS 6417
 MS 1379
 MS 5452
 MS 2134
 MS 6661
 MS 4855
 MS 6985
 MS 3755
 MS 5333
 MS 6524
 MS 2170
 MS 7077
 MS 6204
 MS 6130
 MS 5985
 MS 4081
 MS 3507
 3 BAH75547 Desulfovibrio magneticus RS-1
 MS 834
 MS 1103
 MS 4497
 MS 3242
 MS 4537
 MS 6904
 MS 6927
 MS 6877
 MS 5329
 MS 4378
 MS 5266
 MS 2872
 MS 1526
 MS 6959
 MS 2971
 MS 5313
 MS 906
 MS 2135
 MS 4169
 U68183 Desulfovibrio gigas
 CP000607 Chlorobium phaeovibrioides DSM 265
 MS 3990
80
75
 MS 1652
 MS 2194
 MS 3527
 MS 5242
 MS 2802
 MS 2579
 MS 1638
 MS 5708
 MS 4202
 MS 3141
 MS 808
 MS 5838
 MS 6916
 MS 6783
 MS 7080
 MS 1788
 MS 3371
 MS 3791
 MS 6846
 AP010904 Desulfovibrio magneticus RS-1
 MS 2927
 MS 3155
 MS 1539
 MS 3784
 MS 6359
 MS 1002
 MS 3684
 MS 4839
 MS 1195
 MS 2402
 MS 5356
 MS 4861
 MS 4501
 MS 1072
 MS 5092
 MS 6600
 MS 6243
 MS 5150
 MS 1317
 AAZ06721 sea water B178
 MS 5919
 MS 3753
 MS 1703
 MS 1076
 MS 509
 MS 2631
 MS 1494
 MS 6447
 MS 6710
 MS 3303
 MS 4443
 MS 1446
 MS 4137
 MS 3075
 MS 4341
 MS 6953
 MS 5335
 MS 5034
 MS 2792
 MS 4897
 AGJR01000007 Desulfobacter postgatei 2ac9
 MS 2923
 MS 6579
 MS 1451
 MS 5490
 MS 3334
88
58
72
50
87
61
69
77
 MS 4105
 MS 1854
 MS 5849
 MS 2507
 MS 6841
 MS 2043
 MS 3564
 MS 464
 MS 2849
 MS 442
 MS 5813
 ABQ50612 Mediterranean Sea H4 RNA E3
 MS 5126
 MS 795
 MS 5844
 MS 4280
 MS 1878
 MS 5811
 MS 1875
 MS 6859
 MS 5504
 ADZZ01000138 Delta proteobacterium NaphS2
 MS 2413
 MS 1952
 MS 5754
 MS 1999
 MS 3840
 MS 1067
 MS 1540
 MS 3190
 MS 3262
 MS 4675
 MS 4856
 MS 2811
 MS 5128
 MS 1231
 MS 4454
 MS 4460
 MS 5096
 MS 5856
 MS 493
 MS 1376
 MS 4233
 MS 3623
 ADO24950 mudflat 600-8cm A10P1
 MS 939
 MS 5901
 MS 6245
 MS 3326
 MS 6292
 MS 3406
 MS 430
 MS 1442
 MS 6683
 MS 4296
 MS 3474
 MS 3016
 MS 4010
 MS 109
 MS 4187
 MS 6455
 MS 4576
 MS 4483
 MS 2019
 MS 5217
 MS 6327
 CP001087 Desulfobacterium autotrophicum HRM2
72
75
52
 MS 4095
 MS 2229
 MS 2422
 MS 1448
 MS 2635
 MS 1329
 MS 4759
 MS 717
 MS 2731
 MS 4164
 MS 3977
 MS 3094
 MS 4298
 MS 4931
 MS 3030
 MS 165
 MS 736
 MS 2103
 MS 6299
 MS 5429
 MS 1704
 MS 4228
 MS 1419
 MS 4236
 MS 3388
 MS 5794
 MS 1026
 MS 6247
 MS 5933
 MS 354
 MS 333
 CP001322 Desulfatibacillum alkenivorans AK-01
 MS 1456
 MS 45
 MS 2381
 MS 3766
 MS 5018
 MS 4368
 MS 3083
 MS 6738
 MS 3649
 MS 1836
 MS 792
 MS 2186
 MS 5554
 CUW00858 Posidonia oceanica sediment MEDIFIX 8.3
 MS 638
 MS 1708
 MS 2873
 MS 6620
 MS 3790
 MS 1364
 ACX68596 mangrove Rhizophora mangle sediment RC21
 MS 4585
 MS 6727
 MS 5234
 MS 6405
 MS 1450
 MS 4922
 MS 1171
 MS 4546
 MS 2560
 MS 4843
 MS 3414
 MS 6467
 MS 752
 CCP42462 sulfidic sediment 4PCSSed
 MS 5980
72
63
92
70
 MS 239
 MS 4046
 MS 5288
 MS 573
 MS 3202
 ALJ51804 North Sea sediment T7-ST7-sed-nifH-16
 MS 2245
 MS 992
 MS 6071
 MS 3852
 MS 5663
 MS 4364
 MS 1061
 MS 6338
 MS 1899
 MS 5082
 MS 6997
 MS 1622
 MS 5746
 MS 2017
 MS 1618
 MS 5555
 MS 5891
 MS 5655
 MS 6486
 MS 4227
 MS 6408
 MS 1105
 MS 4020
 MS 2478
 MS 5601
 MS 2434
 MS 1849
 MS 2785
 MS 958
 MS 5903
 AHN50574 MD soil MDE amb 26h8
 MS 4096
 MS 5015
 MS 311
 MS 4167
 MS 1097
 AF414637 oligotrophic tropical seagrass bed SD2
 MS 3421
 MS 2688
 MS 6380
 MS 1826
 MS 953
 MS 1991
 MS 153
 MS 4281
 MS 1161
 MS 2823
 MS 1348
 MS 2352
 MS 3993
 MS 6280
 MS 2157
 MS 2155
 MS 3254
 MS 3992
 MS 2315
 MS 6529
 MS 4088
 MS 6218
 MS 2519
 MS 1084
 MS 3657
 MS 3653
 MS 5411
 MS 2912
 MS 3941
 MS 3693
 MS 385
 MS 126
 MS 6443
 MS 7035
 MS 3373
 MS 2790
 MS 4242
 MS 79
 MS 6533
 MS 4072
 MS 3923
 MS 6696
 MS 1058
 MS 2274
 MS 5297
 MS 2674
 MS 4603
 MS 1477
 MS 2977
 MS 4056
 MS 7121
 MS 1316
 MS 4355
 MS 3319
 MS 1107
 MS 1041
 MS 3192
 MS 3568
 MS 2350
 MS 5876
 MS 3797
 MS 7010
 MS 168
 MS 4995
 MS 6949
 3 ACN14079 Desulfobacterium autotrophicum HRM2
 MS 3100
 MS 4810
 MS 778
 MS 1694
 MS 718
 MS 2561
 MS 919
 AAS57677 Spartina alterniflora plant stem S 1B5
 MS 5649
 MS 3833
 MS 5038
 MS 6074
 MS 5743
 MS 1820
 MS 3899
 MS 1987
 MS 1813
 MS 6189
 MS 3553
 MS 5542
 MS 706
 MS 2473
 MS 6539
 MS 3249
 MS 4533
50
 MS 1650
 MS 2294
 MS 6387
 MS 5564
 MS 2617
 MS 4721
 MS 5420
 MS 2967
 CP000027 Dehalococcoides ethenos 195
 MS 1356
 MS 2528
 MS 2132
 MS 5477
 MS 6499
 MS 5536
 MS 4322
 MS 2830
 MS 6709
 MS 6457
 MS 859
 MS 3336
 MS 5469
 MS 4000
 MS 114
 MS 4222
 MS 5230
 MS 5873
 MS 2273
 MS 3850
 MS 4500
 MS 2298
 MS 423
 AHN51107 MD soil MDE elv 38h8
 MS 4173
 MS 6787
 MS 3240
 MS 1906
 MS 897
 MS 5456
 MS 3321
 MS 2805
 MS 6371
 MS 935
 MS 5625
 MS 6973
 AFD03677 marine sediment mesocosms 78
 MS 3533
 MS 2526
 MS 5400
 MS 2951
 MS 3574
 MS 4572
 MS 416
 MS 3500
 CP002117 Methanoplanus petrolearius DSM 11571
 MS 4285
 MS 6434
 MS 2321
 MS 4334
 MS 3145
 MS 2039
 MS 4957
 MS 3216
 MS 2885
 MS 3207
 MS 3182
 ADD49329 marine microbial mats 08-III.97
66
 MS 131
 MS 6475
 MS 7109
 CP001100 Chloroherpeton thalassium ATCC 35110
 MS 6055
 MS 121
 MS 3580
 MS 6679
 MS 4488
 MS 1066
 AE006470 Chlorobium tepidum TLS
 MS 7122
 MS 6979
 MS 4440
 MS 6882
 MS 6872
 MS 4449
 MS 1685
 MS 6344
 MS 6079
 MS 1075
 MS 1903
 MS 708
 MS 4558
 MS 477
 MS 5958
 CP001108 Prosthecochloris aestuarii DSM 271
 MS 1196
 CP001666 Clostridium ljungdahlii DSM 13528
 MS 4644
 MS 6635
 CP001099 Chlorobaculum parvum NCIB 8327
 MS 721
 MS 6943
 MS 2888
 MS 5513
 MS 3355
 MS 1981
 MS 485
 MS 3656
 MS 448
 MS 1956
 MS 6484
 MS 1637
 MS 6729
 MS 1300
 MS 2128
 MS 6385
 MS 4349
 MS 5184
 MS 5895
 MS 2668
 MS 5446
 MS 969
 MS 2938
 MS 1404
 MS 1733
 MS 5042
 AP010656 Candidatus Azobacteroides pseudotrichonymphae genomovar CFP2
 MS 2456
 MS 3605
 MS 5236
 MS 4053
 MS 1881
 MS 6831
 MS 2750
65
85
51
93
88
67
 MS 6549
 MS 6918
 MS 1554
 MS 3494
 MS 3109
 MS 3823
 MS 2815
 MS 5152
 MS 3557
 MS 6222
 MS 5768
 MS 6887
 MS 5255
 MS 3727
 MS 3965
 MS 2566
 MS 5908
 MS 1431
 ADT89891 South China Sea uncultured marine bacterium A0-0M-11
 MS 4891
 MS 3503
 MS 4342
 MS 5619
 MS 5180
 MS 5487
 MS 3236
 MS 1592
 MS 5142
 MS 802
 MS 3520
 MS 5874
 CP000673 Clostridium kluyveri DSM 555
 MS 4637
 MS 1676
 CP002345 Paludibacter propionicis WB4
 MS 3604
 MS 1883
 MS 5183
 MS 3679
 MS 5925
 MS 361
 MS 2233
 MS 6136
 MS 4818
 MS 4566
 MS 1900
 MS 2105
 MS 1894
 MS 4399
 MS 6496
 MS 5006
 MS 686
 MS 5915
 CP002028 Thermincola sp. JR
 MS 6407
 MS 5274
 NC15589 Desulfotomaculum ruminis DSM 2154
 MS 4097
 AEVP01000028 Desulfotomaculum nigrificans DSM 574
 MS 1915
 MS 6821
 CP002629 Desulfobacca acetoxidans DSM 11109
 MS 2255
 MS 3799
 MS 5607
 MS 1893
 MS 6249
 MS 5023
82
50
 MS 2297
 MS 2712
 MS 5515
 MS 4264
 MS 6556
 MS 5069
 MS 5358
 MS 5568
 MS 2203
 MS 6781
 MS 6960
 MS 3957
 MS 359
 MS 682
 X07475 Clostridium pasteurianum
 MS 2327
 MS 5292
 MS 5076
 X07472 Clostridium pasteurianum
 MS 652
 MS 1430
 CP001338 Methanosphaerula palustris E1-9c
 MS 2765
 MS 5733
 MS 6441
 ACPD01000046 Clostridium cellulovorans 743B
 ADVF01000044 Clostridium lentocellum DSM 5427
 MS 2943
 MS 5731
 MS 6677
 MS 2219
 MS 737
 CP000724 Alkaliphilus metalliredigens QYMF
 ACXX01000015 Clostridium papyrosolvens DSM 2782
 MS 2616
 MS 4042
 MS 1700
 MS 3890
 MS 4305
 CP000721 Clostridium beijerinckii NCIMB 8052
 MS 1220
 MS 6471
 MS 1800
 APP90039 paddy soil NX ST nifH213
 MS 3831
 MS 1686
 MS 4030
 MS 4862
 MS 5836
 MS 1031
 MS 6840
 MS 4473
 MS 1481
 ACJN01000010 Desulfonatronospira thiodismutans ASO3-1
 MS 3417
 MS 1832
 MS 4423
 MS 3857
 MS 2618
 MS 6870
 MS 3675
 MS 5052
 MS 5702
 MS 1461
 MS 4288
 MS 3433
62
94
75
71
52
94
 MS 3041
 MS 1073
 MS 6634
 MS 2670
 MS 5524
 MS 5651
 MS 3979
 MS 6818
 MS 1949
 MS 4825
 MS 1032
 CP001101 Chlorobium phaeobacteroides BS1
 MS 6958
 MS 6167
 MS 6915
 MS 6731
 MS 3673
 MS 3983
 MS 4320
 MS 4589
 MS 5582
 MS 6094
 MS 2846
 MS 2060
 ADM51366 Salt Marsh rhizosphere JPS8-5
 MS 5107
 MS 5659
 AHN50562 MD soil MDE amb 26g2
 MS 2136
 MS 3177
 MS 2489
 MS 5007
 AAZ77198 alt pond microbial mat nif1003308L15
 MS 4889
 MS 4998
 MS 2738
 MS 6263
 MS 5962
 MS 5043
 MS 4261
 MS 5132
 MS 6726
 MS 4025
 MS 6056
 MS 6058
 MS 2804
 ACOK01000116 Ruminococcus flavefaciens FD-1
 AAQF01000015 Delta proteobacterium MLMS-1
 MS 3015
 MS 3022
 MS 6021
 MS 6775
 MS 3378
 MS 6420
 MS 4517
 MS 2477
 MS 3787
 MS 4648
 MS 5890
 MS 3522
 MS 5680
 MS 604
 MS 1080
 MS 5438
 MS 3514
 MS 5280
 MS 2983
89
56
95
62
88
60
65
57
53
 MS 4714
 MS 2624
 MS 2156
 CP000478 Syntrophobacter fumaroxidans MPOB
 MS 5197
 MS 2291
 MS 5271
 MS 6469
 MS 1088
 MS 4621
 MS 4691
 MS 2871
 MS 776
 MS 2331
 MS 5176
 MS 2159
 MS 3695
 ABD74347 smooth cordgrass rhizosphere N069
 MS 5273
 MS 5272
 MS 5569
 MS 2310
 MS 3324
 MS 5121
 MS 4748
 MS 4250
 MS 7038
 MS 3805
 MS 4674
 MS 2573
 MS 5694
 MS 6869
 ABD74383 smooth cordgrass rhizosphere N105
 MS 6602
 MS 5221
 MS 4987
 MS 1689
 MS 3201
 AAZ77253 hypersaline microbial mat nif1003301M02
 MS 3248
 MS 6945
 MS 5279
 MS 5233
 MS 862
 MS 4972
 MS 3366
 MS 1125
 MS 3439
 MS 6500
 MS 5722
 MS 6966
 MS 2179
 MS 2360
 MS 5294
 MS 6830
 ARA12279 Acidic Peatland SP
 MS 6282
 AKS11305 Huanghai Sea and Bohai Sea sediment B42 35
 AGP75720 uncultured coral associated bacterium XSZ86
 MS 254
 MS 7099
 MS 4826
 ALJ51661 North Sea sediment T7-ST1-sed-nifH-38
 MS 2029
 MS 6745
 MS 3930
 MS 5138
53
76
54
 MS 3581
 MS 5147
 MS 4568
 MS 2235
 MS 5780
 MS 4146
 CP002364 Desulfobulbus propionicus DSM 2032
 MS 2127
 MS 1527
 MS 3717
 MS 1735
 MS 1398
 MS 4238
 CP001649 Desulfovibrio salexigens DSM 2638
 MS 1714
 MS 6168
 MS 4511
 MS 3126
 MS 2076
 MS 3526
 MS 1363
 MS 3855
 MS 3310
 DS990592 Verrucomicrobiae bacterium DG1235
 MS 4494
 BAV19223 Seto Inland Sea environmental sample H3-10m-Mar 4
 ANJ77629 coral associated OTU 930
 MS 1413
 MS 4393
 MS 6560
 MS 4668
 MS 6827
 MS 764
 MS 3956
 MS 3165
 MS 5729
 MS 3390
 AHN50825 MD soil MDE elv 11g2
 MS 3312
 MS 2638
 MS 2988
 MS 5639
 MS 4199
 MS 5157
 MS 1727
 MS 5327
 CP001940 Desulfurivibrio alkaliphilus AHT2
 MS 5678
 MS 3508
 MS 5133
 MS 2774
 MS 915
 MS 4780
 MS 4534
 MS 3785
 MS 1269
 MS 1781
 MS 2053
 AKS11838 pearl river estuary F412 08
 MS 4144
 MS 4106
 MS 1337
 MS 6080
 MS 3647
 MS 5590
 MS 3034
 MS 5775
60
79
79
83
75
99
50
 MS 6809
 MS 5862
 AE001437 Clostridium acetobutylicum ATCC 824
 MS 6252
 MS 3110
 MS 1593
 MS 241
 MS 3074
 MS 3733
 MS 4997
 MS 6262
 MS 6545
 MS 2379
 MS 3680
 MS 6782
 MS 3634
 MS 6330
 MS 6302
 MS 3961
 EDY82020 Verrucomicrobiae bacterium DG1235
 AJP08283 Dongkemadi glacier foreland 16-89
 MS 6646
 MS 4908
 MS 818
 MS 5389
 MS 1874
 MS 5766
 MS 6803
 MS 3815
 AIO06778 intertidal microbial mat 1 A A01
 MS 4122
 AKV16136 Rongcheng Bay surface sediment FC16
 MS 1773
 MS 3935
 ADO24992 mudflat uncultured bacterium 0 07 -8cm B07P1
 MS 6052
 MS 2663
 MS 4389
 MS 2065
 MS 4177
 MS 5959
 MS 4875
 MS 5997
 MS 4840
 MS 3510
 AAO67690 Choptank River CB914H7
 MS 2919
 MS 2998
 MS 6866
 MS 5449
 AAL06271 RNCERR microbial mat BS0799 2130 R13
 MS 1349
 MS 1963
 MS 3563
 MS 7134
 MS 2642
 MS 494
 MS 6658
 MS 3347
 CM001160 Desulfovibrio africanus str. Walvis Bay
 MS 892
 MS 2899
 MS 4581
 MS 6077
 MS 5499
 MS 1607
 MS 4887
 AHN51033 MD soil MDE elv 20f7
61
51
90
55
74
 MS 3148
 CP002116 Spirochaeta smaragdinae DSM 11293
 MS 3625
 MS 4474
 MS 2845
 MS 4623
 MS 3948
 MS 4407
 MS 4220
 ADO24982 mudflat 0 07 -8cm A05P1
 MS 4715
 MS 319
 MS 2979
 MS 2487
 MS 3178
 MS 5633
 MS 6461
 MS 5319
 DS989843 Coleofasciculus chthonoplastes PCC 7420
 MS 98
 MS 4218
 MS 6893
 AHN50487 MD soil uncultured bacterium MDE amb 23f1
 MS 2650
 MS 3706
 MS 1751
 MS 5372
 AKS11699 pearl river estuary P07 12
 MS 6541
 MS 6345
 MS 1667
 MS 4641
 MS 5489
 3 ADU62037 Desulfovibrio aespoeensis Aspo-2
 MS 87
 MS 1357
 MS 5630
 MS 894
 MS 2611
 AFQ33568 contaminated soil BG-2 BG2.19
 MS 4110
 MS 4090
 MS 6697
 MS 3063
 MS 1882
 MS 5826
 ASD49984 Cochin Estuary CE13-N16
 MS 2960
 MS 609
 MS 5833
 WP 015725710 Desulfobulbus propionicus
 AMX23525 dried sludge 55
 MS 320
 MS 6551
 MS 246
 MS 5033
 AAN63084 marine microbial mat BS0997 D02
 MS 2771
 MS 6174
 MS 1808
 MS 5056
 ADDI01000001 Desulfovibrio aespoeensis Aspo-2
 MS 765
 MS 6116
 MS 5752
 MS 6012
 AEU12183 Acropora millepora mucus DM1-14
 MS 6734
50
98
52
58
56
50
70
59
78
63
82
51
0.1
Figure S1 Phylogenetic tree of the NifH sequences (all the OTUs) constructed with the neighbor-joining method. Bootstrap values higher than 50% of 100 resamplings are shown near the corresponding nodes. The chlorophyllide reductase iron protein subunit BchX sequences from Rhodobacter capsulantus and R. sphaeroides were used as outgroup.
