# Supplementary material for: Diversity and Structure of Diazotrophic Communities in Mangrove Rhizosphere, Revealed by High-Throughput Sequencing
Source: Front Microbiol. 2017 Oct 18;8:2032. doi: 10.3389/fmicb.2017.02032 (PMC5651520; doi:10.3389/fmicb.2017.02032)
Supplement: Supplementary file 2 [file Data_Sheet_1.docx]

Supplementary Material

Diversity and structure of microbial diazotrophic communities in a mangrove rhizosphere as revealed by high throughput sequencing

Yanying Zhang, Qingsong Yang, Juan Ling, Joy D. Van Nostrand, Zhou Shi, Jizhong Zhou, Junde Dong*

*** Correspondence:** Junde Dong:dongjd@scsio.ac.cn

# Supplementary Tables

## Supplementary Tables

Table S1 Phylogenetic composition of *nifH* genes at both OTU and sequence level (n=6) (expressed as mean value and standard error, SE).

|  | *R. apiculata* | | *A. marina* | | *C. tagal* | |
| --- | --- | --- | --- | --- | --- | --- |
|  | OTUs | *Sequences* | OTUs | *Sequences* | OTUs | *Sequences* |
|  | (mean±SE) | | (mean±SE) |  | (mean±SE) |  |
| Cluster I | 93.16±1.07 | 5209.5±147.23 | 91.17±1.48 | 4147.33±134.24 | 103.67±6.84 | 3363.83±98.66 |
| Cluster II | 0.83±0.16 | 2.83±0.59 | 0.5±0.14 | 2.17±0.41 | 1.17±0.07 | 1.83±0.19 |
| Cluster III | 517.83±9.89 | 6787.67±146.95 | 463.33±9.43 | 7840±133.22 | 507.67±10.22 | 8634±98.69 |
| Cluster IV | 0 | 0.08±0.01 | 2±0.46 | 10.5±2.66 | 0.33±0.09 | 0.33±0.09 |

Table S2 The analysis of statistical significant differences based on phylogenetic composition at both OTU and sequence level (RA, *R. apiculata*, AM, *A. marina*, CT, *C. tagal*).

|  | Cluster I | Cluster II | Cluster III | Cluster IV |
| --- | --- | --- | --- | --- |
|  | *P*  *t-test* | *P*  *t-test* | *R*  *t-test* | *P*  *t-test* |
| *OTUs* |  |  |  |  |
| RA vs AM | 0.889 | 0.472 | 0.131 | *0.049* |
| RA vs CT | 0.470 | 0.472 | 0.770 | 0.726 |
| AM vs CT | 0.391 | 0.160 | 0.214 | 0.095 |
| Sequences |  |  |  |  |
| RA vs AM | *0.030* | 0.662 | *0.031* | 0.067 |
| RA vs CT | *0.001* | 0.514 | *0.001* | 0.951 |
| AM vs CT | 0.098 | 0.827 | 0.093 | 0.075 |

Significant differences (P < 0.05) are indicated in italics.
